# Effects of chemical combinations on the parthenogenetic activation of mouse oocytes

**DOI:** 10.3892/etm.2013.1018

**Published:** 2013-03-19

**Authors:** BAO-SHENG HAN, JUN-LING GAO

**Affiliations:** 1 Department of Reproduction and Genetics, Maternity and Child Health Care Hospital, Tangshan, Hebei 063000, P.R. China; 2 Department of Histology and Embryology, North China Coal Medical University, Tangshan, Hebei 063000, P.R. China

**Keywords:** mouse, oocyte, parthenogenetic activation, combined activation

## Abstract

The aim of this study was to identify an optimal method for the parthenogenetic activation of mouse oocytes. Ethanol (EH), strontium chloride (SrCl_2_) and ionomycin calcium salt were each combined with cytochalasin B to induce the parthenogenetic activation of CD-1^®^ mouse oocytes. Among the EH combination groups, the blastocyst formation and hatching rates of the group that was activated with EH and CB for 5 min were significantly higher compared with those of the groups that were activated for 7 and 10 min (P<0.05). Among the SrCl_2_ combination groups, the blastocyst formation and hatching rates of the group that was activated with SrCl_2_ and CB for 30 min were significantly higher compared with those of the groups that were activated for 1 and 2 h (P<0.05). Among the ionomycin calcium salt combination groups, the blastocyst formation and hatching rates of the group that was activated with ionomycin and CB for 3 min were higher compared with those of the groups that were activated for 5 and 7 min (P<0.05). Compared with the other two combinations, the experimental indicators of the EH combination groups were notably superior (P<0.05). For combined activation, simultaneous activation with two substances was significantly more effective than successive activation (P<0.05). For combined activation with EH and cytochalasin B in mouse oocytes, 5 min of parthenogenetic activation had significant advantages with regard to cleavage, blastocyst formation and blastocyst hatching rates. In addition, the activation rate of combined activation was higher than that of single activators. For combined activation, the simultaneous application of two activators has a superior effect.

## Introduction

At present, embryonic stem cells for research are derived from fertilized embryos. However experimental studies on human oocytes and embryos are limited by numerous religious, ethical and moral considerations. Therefore, there are few reports and detailed studies. As embryonic stem cells formed by parthenogenetic activation have the same totipotency and proliferation as those formed by sperm-egg fusion, they are able to directionally differentiate and develop. Consequently, parthenogenetic activation technology has become a focus of reproductive biology.

The aim of parthenogenetic activation technology is to simulate the fertilization process by artificial stimulation to activate oocytes under non-sperm conditions. Female gametes generate embryos and the embryos continuously develop, mature and generate posterior polarity (1). Parthenogenetic activation technology provides embryo replacement resources for experimental studies concerning the interaction of male and female gametes and the initial development mechanism of embryos. It may also provide an improved understanding of physiological mechanisms such as the signal coupling process and activation kinetics of oocytes and recovery of cell cycle arrest in the sperm-egg fusion process. Additionally, mature oocyte activation *in vitro* is an essential condition for the activation of embryo reconstruction by nuclear transplantation (2–4). This technology is also an important technique for establishing embryonic stem cell lines (5) and it may be used in gene therapy, organ transplantation, tissue repair, disease treatment and other medical fields.

The animals used for parthenogenetic activation studies include rats, rabbits, cattle, sheep, pigs, horses and monkeys. Studies suggest that for different animal species or germ lines from the same species, the same activation conditions cause variable results. It has been suggested that rabbits are particularly suitable for use in studies of parthenogenetic activation technology (6). Although there have been studies where live embryos of apomixic mice were born and developed into mature individuals with reproductive capacity (7) and mouse embryonic stem cell lines have been established by parthenogenetic activation technology (8,9), parthenogenetic mouse embryos rarely develop to the full term to be born. Therefore, study data concerning parthenogenetic activation technology for mouse oocytes is extremely limited at present (5).

Currently, the key problems restricting and affecting the subsequent development of embryos formed by parthenogenetic activation are that the parthenogenetic activation methods are not well developed, blastocyst formation rates are low, morphologies are poor and the majority of formed blastospheres have no potential for continuous development. Therefore, the aims of this field of research are to investigate and optimize the parthenogenetic activation method, as well as the blastocyst formation proportion and quality, to provide high-quality blastospheres for subsequent studies.

At present, there are two main types of parthenogenetic activation methods: physical methods and chemical methods. The former includes mechanical, temperature and electrical stimulation, while the latter includes enzymes, hyper- or hypotonic solution environments and ion and protein synthesis inhibitor treatments. Various activation methods affect the further developmental potential of embryos. A long-term research goal has been the identification of the optimum method for parthenogenetic activation which results in improved or complete oocyte gene recombination to achieve the largest proportion of activated cells. Additionally, the activated cells should develop to the embryo stage and on to continuous maturation (5). At present, studies suggest that a single activation method (physical or chemical method) is inferior to combined activation methods (10,11).

In the present study, ethanol (EH), strontium chloride (SrCl_2_) and ionomycin calcium salt were each combined with cytochalasin B (CB) to induce the parthenogenetic activation of cesarean-derived-1^®^ (CD-1^®^) mouse oocytes (Beijing Vitalriver Laboratory Animal Technology Co., Ltd., Beijing, China) and the cleavage, overspeed cleavage (cleavage rate is faster than that of the normal zygote, blastomere is above 6), blastocyst formation and blastocyst hatching rates were measured in order to identify the optimal activation method and provide further standard experimental methods and data concerning parthenogenetic activation, embryo cloning and nuclear transplantation reconstruction embryo activation in mice.

## Materials and methods

### Experimental animals

Female CD-1^®^ mice (age range, 10–12 weeks; bodyweight range, 20–25 g) were bred under artificial temperature control (22–24°C) and lighting conditions (6:00–20:00, light; 20:00–6:00, dark). The mice were able to drink water and eat freely. The present study was performed in strict accordance with the recommendations of the Guide for the Care and Use of Laboratory Animals of the National Institutes of Health. The animal use protocols were reviewed and approved by the Institutional Animal Care and Use Committee (IACUC) of the Maternity and Child Health Care Hospital (Tangshan, China).

### Oocyte collection

At 20:00, 10 IU urinary human menopausal gonadotropin (HMG) was administered to the mice via intraperitoneal injection. After 48 h, 10 IU human chorionic gonadotrophin (hCG) was administered via intraperitoneal injection. At 14–16 h (10:00–12:00) after the hCG injection, the abdominal cavity of each mouse was cut open to remove the oviduct and the ovoplasmon was removed under a microscope and transferred for treatment into modified human tubal fluid (MHTF) containing 80 IU/ml hyaluronidase for 1 min. The resulting mixture was blown to remove granulosa cells and washed repeatedly with MHTF. Normal-morphology stage MII oocytes, containing the first polocyte, were selected and stored in an incubator for use. At 18 h after injecting hCG (2), (14:00 the same day), the activation experiment was performed.

### Experimental groups

EH, SrCl_2_ and ionomycin calcium salt (ionomycin) were each combined with CB to give 3 combinations in total. For the first combination (EH and CB), the final concentration of EH was 7% and of CB was 5 *μ*g/ml (10). This combination was further divided into 6 groups: group 1 (activated with EH plus CB for 5 min), group 2 (activated with EH plus CB for 7 min) (10), group 3 (activated with EH plus CB for 10 min), group 4 (first activated with EH for 7 min, then continuously activated with CB for 7 min), group 5 (activated with CB for 7 min) and group 6 (EH was replaced with an equivalent amount of culture liquid without CB and this group was used as the control group). For the second combination (SrCl2 and CB), the final concentration of SrCl_2_ was l0 mmol/l (10) and of CB was 5 *μ*g/ml. This combination was further divided into 6 groups: group 1 (activated with SrCl_2_ plus CB for 30 min), group 2 (activated with SrCl_2_ plus CB for 1 h), group 3 (activated with SrCl_2_ plus CB for 2 h) (11), group 4 (first activated with SrCl_2_ for 1 h, then continuously activated with CB for 7 min), group 5 (activated with CB for 7 min) and group 6 (SrCl_2_ was replaced with an equivalent amount of culture liquid and this group was used as the control group). For the third combination (ionomycin and CB), the final concentration of ionomycin was 5 *μ*mol/l (12) and of CB was 5 *μ*g/ml. This combination was further divided into 7 groups: group 1 (activated with ionomycin plus CB for 3 min), group 2 (activated with ionomycin plus CB for 5 min), group 3 (activated with ionomycin plus CB for 7 min), group 4 (first activated with ionomycin for 5 min, then continuously activated with CB for 5 min), group 5 (activated with CB for 5 min), group 6 [treated with MHTF containing 0.1% dimethyl sulfoxide (DMSO) for 5 min] and group 7 (ionomycin was replaced with an equivalent amount of culture liquid and this group was used as the control group).

### Oocyte activation and embryo culture in vitro

All the chemical reagents for activating oocytes were prepared with MHTF containing 10% serum substitute (SSS). Ionomycin calcium salt was dissolved in MHTF containing 0.1% DMSO (10% SSS). The activation dish (35-mm dish, 50 *μ*l/drop) was prepared and balanced in a CO_2_ incubator for ∼1 h prior to use. The stage MII oocytes were randomly distributed into the experimental and control groups, then activated in the CO_2_ incubator. In this study, a total of 296 mice were used for 26 batches of activation tests (10–13 mice/batch) and a total of 7,006 stage MII oocytes with normal morphologies were collected. After activation was complete, conventional culture was performed and the culture liquid was replaced. The procedures complied with the human embryo culture technique specification (13). At 16–18 h (afternoon of day 1) of activation processing, the activation results were observed. The cleavage and overspeed cleavage, blastocyst formation and blastocyst hatching rates were observed and measured on day 2, day 4 and day 5, respectively. The activation culture was maintained in the incubator for a total of 120 h.

### Morphological observations

Observations were performed under an inverted microscope to record the status of the first polocyte discharge and pronucleus formation. If an oocyte exhibited a pronucleus or cleavage (containing cytoblast), it was considered to be activated, while if the oocyte membrane was broken, it was considered to be a dead cell. If an oocyte had no evident changes, it was regarded to be a regenerated cell.

The oocytes were recorded as 1 pronucleus (1 PN), 2 pronuclei (2 PN), overspeed cleavage and other (3 pronuclei and other situations) according to the pronucleus and polocyte discharge status during activation.

### Natural fertilization group

Following the administration of 10 IU hCG to the mice via intraperitoneal injection, male and female mice were caged in a 1:1 ratio. According to the activation procedures, the oviducts and ovoplasmon (fertilized) were removed and cultured in a CO_2_ incubator. Activated embryos were observed and counted simultaneously.

### Hematoxylin and eosin (H&E) staining

Parthenogenetically activated and naturally fertilized embryos were selected and placed on slides and the slides were dried naturally. According to the H&E staining procedure, fixation, staining and mounting were performed successively and the observations, analysis and image acquisition were carried out under a microscope.

### Statistical analysis

The cleavage, overspeed cleavage, blastocyst formation and blastocyst hatching rates were calculated according to the following formulae: Cleavage rate (%) = cleavage count/oocyte (MII) count; overspeed cleavage rate (%) = overspeed cleavage count/cleavage count; blastocyst formation rate (%) = blastocyst count/cleavage count; blastocyst hatching rate (%) = blastocyst hatching count/cleavage count. For the experimental data, χ^2^ tests with SPSS 11.5 software were used for statistical analysis. P<0.05 was considered to indicate a statistically significant difference.

## Results

### Combined application of EH and CB

**Figure 1:**
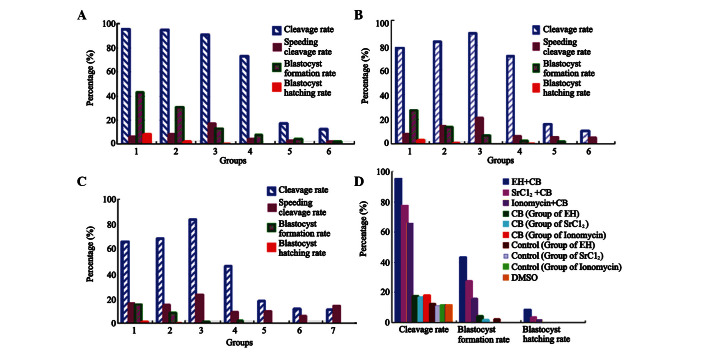
Activation results of combined application of (A) EH and CB; (B) SrCl_2_ and CB; (C) ionomycin with CB; (D) comparisons of relevant data among the three chemical combination methods. EH, ethanol; CB, cytochalasin B; SrCl_2_, strontium chloride.

**Figure 2:**
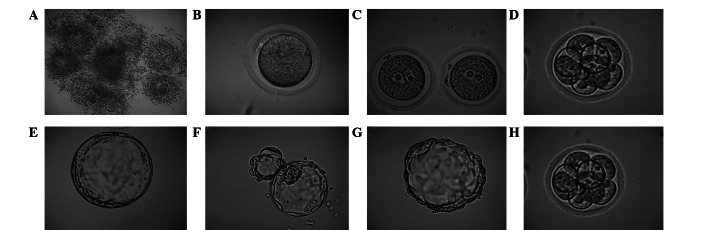
*In vitro* development of parthenogenetic embryos: (A) Oocyte cumulus-corona radiata complex under a phase-contrast microscope (magnification, ×200); (B) oocytes of the first polocyte following enzyme treatment (magnification, ×400); (C) ovum with 2 pronuclei (magnification, ×400); (D) 8 blastocytes (magnification, ×400); (E) blastosphere in expansion stage, (magnification, ×400); (F) blastospheres being hatched; (G) hatched blastosphere (magnification, ×400) (H) there were an increased number of segments in the zona pellucida (magnification, ×400).

Among groups 1, 2 and 3, there were no significant differences in the the cleavage rate (95.5, 94.4 and 90.8%, respectively; P>0.05), while there were significant differences in the blastocyst formation (43.3, 31.0 and 13.1%, respectively) and blastocyst hatching rates (8.62, 2.46 and 0.25%, respectively; P<0.05). Among them, the activation effectiveness of group 1 was the highest. Between groups 2 and 4, there were significant differences in the cleavage (94.4 vs. 73.0%), blastocyst formation (31.0 vs. 7.83%) and blastocyst hatching rates (2.46 vs. 0%; P<0.05). There were significant differences between the cleavage, blastulation and blastocyst hatching rates of the combined activation groups (groups 1–4) and those of group 5 (17.6, 4.28 and 0%, respectively) and of the control group (12.3, 2.27 and 0%, respectively; P<0.05). In addition, the overspeed cleavage rate of group 3 was significantly higher than that of any other group (P<0.05; Table I and Figs. 1A and 2A–G).

### Combined application of SrCl_2_ and CB

Among groups 1, 2 and 3, there were no significant differences in the cleavage rate (78.0, 82.7 and 89.7%, respectively; P<0.05). However, there were significant differences in the blastocyst formation (27.6, 14.1 and 7.25%, respectively) and blastocyst hatching rates (3.61, 1.19 and 0%, respectively; P<0.05). Among them, the activation effectiveness of group 1 was the highest. Between groups 2 and 4, there were significant differences in the cleavage (82.7 vs. 71.2%) and blastocyst formation rates (14.1 vs. 2.53%; P<0.05), while there were no significant differences in the blastocyst hatching rate (1.19 vs. 0.36%; P>0.05). There were significant differences in the cleavage, blastocyst formation and blastocyst hatching rates between the combined activation groups (groups 1–4) and group 5 (16.5, 1.92 and 0%, respectively) and the control group (10.9, 0 and 0%, respectively; P<0.05). In addition, the overspeed cleavage rate of group 3 was significantly higher than that of any other group (P<0.05; Table II and Fig. 1B).

### Combined application of ionomycin and CB

Among groups 1, 2 and 3, there were significant differences in the cleavage (65.9, 68.0 and 83.3%, respectively; P<0.05), blastocyst formation (15.8, 8.88 and 1.34%, respectively) and blastocyst hatching rates (1.66, 0 and 0%, respectively; P<0.05). Among them, group 1 had the lowest cleavage rate but the highest blastocyst formation rate. Between groups 2 and 4, there were significant differences in the cleavage (68.0 vs. 46.4%) and blastocyst formation rates (8.88 vs. 2.53%; P<0.05), while there were no significant differences in the blastocyst hatching rate (0.39 vs. 0%; P>0.05). With regard to comparisons of the combined activation groups (groups 1–4) with groups 5 and 6 and the control group, there were significant differences in the cleavage rate (P<0.05). There were significant differences in the blastocyst formation and blastocyst hatching rates (P<0.05) only between groups 1 and 2 and groups 5 and 6 and the control group, while there were no significant differences between groups 3 and 4 and these 3 groups (P>0.05). In addition, the overspeed cleavage rate of group 3 was higher than that of any other group (P=0.05; Table III and Fig. 1C).

### Comparisons of relevant data among the three chemical combination methods

Among the three chemical combination methods, the first combination (combined application of EH and CB) was the most effective and, for the EH, SrCl_2_ and ionomycin combinations, there were significant differences between cleavage (95.5, 78.0 and 65.9%, respectively), blastocyst formation (43.3, 27.6 and 15.8%, respectively) and blastocyst hatching rates (8.62, 3.61 and 1.66%, respectively; P<0.05). In the simple CB groups, there were no significant differences in the cleavage (17.6, 16.5 and 18.0%, respectively), blastocyst formation (4.28, 1.92 and 0%, respectively) and blastocyst hatching rates (0, 0 and 0%, respectively; all P>0.05) among the three chemical combination methods. In the control groups, there were no significant differences in the cleavage (12.3, 10.9 and 11.4%, respectively), blastocyst formation (2.27, 0 and 0%, respectively) and blastocyst hatching rates (0, 0 and 0%, respectively) among the three chemical combination methods (all P>0.05). With regard to comparisons of the simple DMSO group with the simple CB groups and control groups, there were no significant differences in any of the indicators (all P>0.05; Table IV and Fig. 1D).

### In vitro development of naturally fertilized embryos

**Figure 3:**
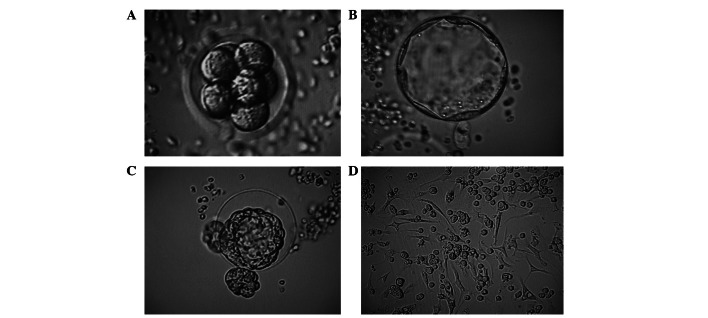
*In vitro* development of normal fertilized embryos: (A) 8 blastocytes; (B) blastosphere in the expansion stage; (C) blastospheres being hatched; (D) granulocytes growing well by attaching to the wall. Magnification, ×400.

It was observed that there were embryos with consistent development rates and culture times in various stages and their blastomere sizes were uniform, their morphologies were normal and there was no segmentation. The zona pellucida was clear and complete. Cell islands of granulocytes growing by attaching to the wall were visible and the cell morphologies became fusiform or stellate. The cells appeared to grow well (Fig. 3).

### H&E staining

**Figure 4:**
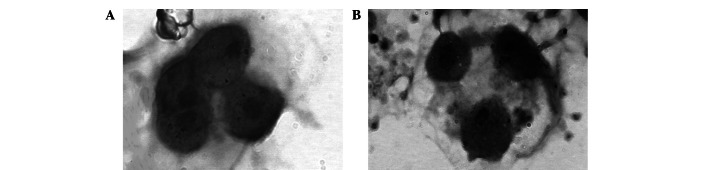
H&E staining: (A) parthenogenetically actived embryos; (B) normally fertilized embryos, dark stained cytoblast and pink cytolymph were visible (magnification, ×400). H&E, hematoxylin and eosin. Magnification, ×200.

The parthenogenetically activated embryos had 4 blastomeres and the blastomere sizes were uniform. Also, bluish violet and basophilic cytoblasts were visible. The cytoblasts had darkly stained nucleoli. The cytolymph was eosinophilic and pink and the zona pellucida was loose around the blastomere. The blastomere morphologies of the naturally-fertilized embryos were similar to those of the parthenogenetically activated embryos. It was observed that there were three blastomeres, the blastomere sizes were slightly nonuniform and the cytoblast had become bluish violet. In the cytoblast, darkly stained nucleoli were present. The cytoplasm was pink and there were scattered granulocytes (Fig. 4).

## Discussion

At present, mouse embryonic stem cell lines are sometimes established using parthenogenetic activation technology (8,9). However, since parthenogenetic mouse embryos rarely develop to the full term to be born, research data on parthenogenetic activation technology in mouse oocytes are extremely limited (5). Mice are one of the most commonly used experimental mammalian animals for genetic research and the genetic background of mice has been extensively studied. Mice have economic advantages due to undergoing early maturation, having strong reproductive capacity and short reproductive cycles and being a convenient animal source. Therefore, mice have considerable advantages for studies of parthenogenetic activation technology.

Under normal physiological conditions, oocytes are activated by signals and factors released by sperm. Endogenous Ca^2+^ levels are elevated and oscillate, activating protein kinase C to cause maturation promoting factor (MPF) levels to decrease, resulting in the initiation of meiosis. Studies suggest that oocyte activation by artificial activation methods causes similar changes to those caused by fertilization. Artificial activation and fertilization are able to induce endogenous Ca^2+^ elevation and oscillation, although the Ca2+ elevation peak caused by sperm is greater. All factors which cause Ca^2+^ oscillation are able to activate oocytes and cloned embryos. A single Ca^2+^ transient elevation is only able to reduce MPF activity and multiple transient Ca^2+^ stimulations are required to maintain low levels of this kinase, which is the precondition for full development of parthenogenetic embryos from mouse oocytes (11). At present, physical factors, such as mechanical, temperature and electric stimulation, and chemical factors, such as enzymes, hyper- or hypotonic solution environments and ion and protein synthesis inhibitor treatments, are known to effectively activate oocytes to various extents.

Studies have shown that when a combination of electrical stimulation and the chemical reagent 6-dimethylaminopurine (6-DMAP) was applied to mouse oocytes, the activation rate and developed blastocyst number were higher than those of a single treatment (5). The high efficiency of combined activation methods has been demonstrated in rabbits, cattle and other animals (6,12). Combined activation is able to cause an irreversible decline in MPF levels and greatly increases the activation rate of oocytes, causing a marked promotion of their late embryonic development. However, different combination methods generate different activation results. Among these methods, the combination of a particular physical method, impulse-type electrical field stimulation, with a chemical method is the most common. Versieren *et al*(10) demonstrated that the combination of electrical stimulation with ionomycin treatment clearly promoted oocyte activation and subsequent embryonic development. However, it has been suggested that this combined activation method is limited since it is difficult to control and regulate the electric field force and pulse frequency vital for the effects of parthenogenetic activation on development. Furthermore, inappropriate stimulation intensity may affect the blastocysts’ subsequent development and the equipment cost is high. Chemical combination methods are also commonly used as the activation method. Since chemical combination methods have the advantages of being convenient and using a wide range of reagent sources, as well as having higher activation rates, they are used in by numerous researchers. Heytens *et al*(11) demonstrated that ionomycin calcium salt was an effective activator and its application concentration was 10 *μ*mol/l. Ma *et al*(14) revealed that when 10 mmol/l SrCl_2_ combined with CB was used for treating oocytes with an ovum age of 18 h for 2.5 h, an improved activation rate was obtained. However, the above studies did not report the blastocyst hatching rate. It appears that the current parthenogenetic activation methods are not well developed since the blastocyst formation proportion is low, morphologies are poor and the majority of formed blastospheres have no potential for continuous development.

In the present study, EH, SrCl_2_ and ionomycin were each combined with CB to perform parthenogenetic activation of CD-1^®^ mouse oocytes and higher cleavage (95.5, 78.0 and 65.9%, respectively), blastocyst formation (43.3, 27.6 and 15.8%, respectively) and blastocyst hatching rates (8.62, 3.61 and 1.66%, respectively) were obtained. Between the combined activation and CB groups, there were significant differences in the cleavage, blastocyst formation and blastocyst hatching rates (all P<0.05). The activation rate of combined activation is higher than that of a simple activator and it appears that the activation rate of the simultaneous activation of two chemical substances is significantly higher than that of successive activation. Furthermore, the combination method is simple, has short activation times and is able to reduce the effects of non-activation factors on oocytes.

EH is an important reagent for parthenogenetic activation. Cuthberston *et al* and Nagai (15,16) used EH to induce the activation of mouse and cattle oocytes in 1981 and 1987, respectively. The activation principle is to generate 1,4,5-inositol triphosphate (IP3) by hydrolyzing 4,5-diphosphoinositide (PIP2) on the oocyte membrane to alter the egg membrane stability, induce intracellular Ca^2+^ signalling increases and increase the release of Ca^2+^ from calcium storage and extracellular Ca^2+^ influx. Therefore, intracellular Ca^2+^ is increased, causing oocyte activation (1). At present, an EH concentration of 7% and action time of 7 min produce the optimum activation rate (11).

CB inhibits the release of the second polocyte after activation, promotes diploid development, prevents chromosome assortment, inhibits cytokinesis and generates a diploid with two pronuclei. CB strengthens the capacity of the oocyte to develop to the blastula stage and also prevents embryonic disintegration (12).

In the present study, 7% EH and 5 *μ*g/m CB were combined according to the literature (10) to treat the oocytes for 5, 7 and 10 min. The results showed that the 5 min group had clear advantages with regard to cleavage, blastocyst formation and blastocyst hatching rates, whereas the 7 and 10 min groups had higher cleavage rates. In addition, the overspeed cleavage rate of the 10 min group was higher and it was observed that there were more segments in the overspeed cleavage embryos and the blastomere morphologies were not uniform (Fig. 2H), indicating that with an increase of activation time, the embryonic development rate was unbalanced and the subsequent development capacity was poor.

Additionally, combinations of SrCl_2_ and ionomycin with CB were each used and the results showed that the cleavage, blastocyst formation and blastocyst hatching rates were lower compared with the combination of CB with EH. It was also visible that there were more segments, blastomere morphologies were not uniform, embryos were incomplete, subsequent development capacity was poor and the death rate was increased.

Among the three chemical combinations, statistical analysis showed that the EH combination had clear advantages with regard to cleavage, blastocyst formation and blastocyst hatching rates. It has been suggested that EH is an effective activator for the parthenogenetic activation of mouse oocytes to replace SrCl_2_ or ionomycin.

Unlike the oocytes that discharge the second polocyte and form diploid zygotes in the natural fertilization process, the karyotypes of parthenogenetically activated embryos are affected by the activation mode, ovum age and experimental conditions and multiple karyotypes such as homogeneous haploids, mosaic haploids and heterozygous diploids may be formed (17). Feng *et al*(18) performed teratological and histological analyses of embryos at 13 days after parthenogenetic activation and implantation and observed that they were normal in morphology. Sex chromosome analysis of parthenogenetic embryos has been performed and it was observed that the majority of parthenogenetic embryo karyotypes were XX and 1 PN embryos had the normal chromosome number. In the present study, H&E staining was used for parthenogenetic and naturally-fertilized embryos and it was observed that the blastomere morphologies of the two types of embryos were consistent and had normal cytoblasts and cytolymphs, indicating that the activated embryos developed normally. The morphologies of the blastospheres hatched by embryos from the EH group were superior and the blastocyst hatching rate was higher, suggesting that the blastospheres had the potential for continuous development.

In the parthenogenetic activation process, the ovulation induction drug dose, ovum maturation, ovum age, culture system and methodological proficiency of the individuals carrying out the experiments all affect the parthenogenetic activation and subsequent development.

Oocytes for experimentation are obtained mainly by promoting ovulation and pregnant mare serum gonadotrophin (PMSG) is usually used to do so. With increases in the medication time and dose, the oocyte number may be increased. However, following parthenogenetic activation, the proportion of oocytes developing into embryos and blastospheres is greatly reduced, suggesting that large doses of PMSG directly affect oocyte quality, fertilization capacity, chromosome structure and spindle function, thus affecting the development potential of mouse embryos (1). In the present study, the dose of HMG used was constant (10 IU/mouse). In further studies, the effects of various doses of ovulation induction drugs on parthenogenetic activation should be investigated.

Parthenogenetic activation studies have shown that oocyte maturation and ovum age are important factors affecting oocyte activation (2) and only mature oocytes may be activated by Ca^2+^ signal increases induced by EH and calcium ionophore, which has been demonstrated in multiple animal species (5). It is generally considered that the higher the maturation, the higher the sensitivity to activator. In addition, the activation rate is increased as the ovum age increases. When the ovum age is >24 h (at 24 h after hCG injection), certain oocytes are possibly naturally activated. However, it has been reported (19) that the parthenogenetic activation rate of the ovum at 14 h is higher than that at 18 h. Clearly, the mechanism of parthenogenetic activation is different from that of fertilization. At present there are few studies concerning the correlation of ovum age with oocyte activation rate and the development mechanism after activation. In the present study, it was required that the mouse ages (bodyweights) were between 10 and 12 weeks (weight, 20–25 g), ovum age was controlled at 18 h after HCG injection and the oocytes were in stage MII with normal morphologies to ensure the reliability and accuracy of the experimental results.

The osmotic pressure of the culture medium is vital for oocyte development and the effects are different for different animal species. For mice, the optimum osmotic pressure of the culture medium is 276 mOsm/l. Our experiences suggest that the effects of the culture system on parthenogenetic activation and subsequent development are signicantly greater. It is required that the CO_2_ concentration is constant in the incubator, temperature and humidity are constant, culture liquid is high-quality and embryonic sequential culture (embryos cultured in different culture media containing different components in various stages) is performed. Also, the experiments should be performed in a 100-level thermostatic platform in a laminar flow room (constant temperature and humidity) to reduce the effects of temperature and humidity variations.

## Figures and Tables

**Table I:** Results of combined application of ethanol (EH) and cytochalasin B (CB).

Group	Oocyte (MII) n	Cleavage n (%)	Overspeed n (%)	Blastulation n (%)	Blastocyst hatching n (%)
1	462	441 (95.5)	29 (6.58)	233 (43.3)	38 (8.62)
2	431	407 (94.4)	33 (8.11)	126 (31.0)	10 (2.46)
3	437	397 (90.8)	69 (17.4)	52 (13.1)	1 (0.25)
4	385	281 (73.0)	12 (4.27)	22 (7.83)	0 (0.00)
5	397	70 (17.6)	3 (2.86)	3 (4.28)	0 (0.00)
6	358	44 (12.3)	1 (2.27)	1 (2.27)	0 (0.00)

Group 1, activated with EH plus CB for 5 min; group 2, activated with EH plus CB for 7 min; group 3, activated with EH plus CB for 10 min; group 4, activated with EH for 7 min, then with CB for 7 min; group 5, activated with CB for 7 min; group 6, treated with culture liquid (control). The final concentration of EH was 7% and of CB was 5 *μ*g/ml. Overspeed, cleavage rate is faster than that of the normal zygote.

**Table II:** Results of combined application of strontium chloride (SrCl_2_) and cytochalasin B (CB).

Group	Oocyte (MII) n	Cleavage n (%)	Overspeed n (%)	Blastulation n (%)	Blastocyst hatching n (%)
1	391	305 (78.0)	26 (8.52)	84 (27.6)	11 (3.61)
2	404	334 (82.7)	51 (15.3)	47 (14.1)	4 (1.19)
3	370	331 (89.7)	71 (21.5)	24 (7.25)	0 (0.00)
4	389	277 (71.2)	18 (6.50)	7 (2.53)	1 (0.36)
5	316	52 (16.5)	3 (5.76)	1 (1.92)	0 (0.00)
6	338	37 (10.9)	2 (5.40)	0 (0.00)	0 (0.00)

Group 1, activated with SrCl_2_ plus CB for 30 min; group 2, activated with SrCl_2_ plus CB for 1 h; group 3, activated with SrCl_2_ plus CB for 2 h; group 4, activated with SrCl_2_ for 1 h, then with CB for 7 min; group 5, activated with CB for 7 min; group 6, treated with culture liquid (control). The final concentration of SrCl_2_ was l0 mmol/l and of CB was 5 *μ*g/ml. Overspeed, cleavage rate is faster than that of the normal zygote.

**Table III:** Results of combined application of calcium ionomycin and cytochalasin B (CB).

Group	Oocyte (M II) n	Cleavage n (%)	Overspeed n (%)	Blastulation n (%)	Blastocyst hatching n (%)
1	366	241 (65.9)	39 (16.2)	38 (15.8)	4 (1.66)
2	381	259 (68.0)	51 (14.7)	23 (8.88)	1 (0.39)
3	359	299 (83.3)	69 (23.1)	4 (1.34)	0 (0.00)
4	334	155 (46.4)	14 (9.03)	2 (2.53)	0 (0.00)
5	283	51 (18.0)	5 (9.80)	0 (0.00)	0 (0.00)
6	297	34 (11.5)	2 (5.88)	0 (0.00)	0 (0.00)
7	308	35 (11.4)	5 (14.3)	0 (0.00)	0 (0.00)

Group 1, activated with ionomycin plus CB for 3 min; group 2, activated with ionomycin plus CB for 5 min; group 3, activated with ionomycin plus CB for 7 min; group 4, activated with ionomycin for 5 min, then with CB for 5 min; group 5, activated with CB for 5 min; group 6, treated with modified human tubal fluid containing 0.1% dimethyl sulfoxide for 5 min; group 7, treated with culture liquid (control). The final concentration of ionomycin was 5 *μ*mol/l and of CB was 5 *μ*g/ml. Overspeed, cleavage rate is faster than that of the normal zygote.

**Table IV:** Comparison of the related data of three chemical combination methods.

Group	Cleavage number (%)	Blastulation number (%)	Blastocyst hatching number (%)
1 (EH + CB)	95.5	43.3	8.62
1 (SrCl_2_ + CB)	78.0	27.6	3.61
1 (Ino + CB)	65.9	15.8	1.66
CB (EH + CB)	17.6	4.28	0.00
CB (SrCl_2_ + CB)	16.5	1.92	0.00
CB (Ino + CB)	18.0	0.00	0.00
Control (EH + CB)	12.3	2.27	0.00
Control (SrCl_2_ + CB)	10.9	0.00	0.00
Control (Ino + CB)	11.4	0.00	0.00
DMSO	11.5	0.00	0.00

Group 1 (EH + CB), activated with EH plus CB for 5 min; group 1 (SrCl_2_ + CB), activated with SrCl_2_ plus CB for 30 min; group 1 (Ino + CB), activated with ionomycin plus CB for 3 min. Groups CB (EH + CB) and CB (SrCl2 + CB), activated with CB alone for 7 min; Group CB (Ino + CB), activated with CB alone for 5 min; Groups Control (EH + CB) and Control (SrCl2 + CB), cultured in the absence of activator for 7 min; Group CB (Ino + CB), cultured in the absence of activator for 5 min; Group DMSO, cultured with DMSO for 5 min. CB, cytochalasin B; EH, ethanol; SrCl_2_, strontium chloride; Ino, ionomycin calcium; DMSO, dimethyl sulfoxide.
